# What a Fish a Frog Is

**Published:** 1893-11

**Authors:** 


### EDITORIAL DEPARTMENT.

F. E. DANIEL, M. D., Editor.
S. E. HUDSON, M. D., Managing Editor.
A. J. SMITH. M. D., Galveston, Associate Editor.

EDITORIAL STAFF:

PROF. J. E. THOMPSON, M. D., Texas Medical College, Galveston; Surgery.
PROF. WM. KEILLER, M. D., Texas Medical College, Galveston; Obstetrics and Gynecology.
PROF. DAVID CERNA, M. D., Texas Medical College, Galveston; Therapeutics.
PROF. A. J. SMITH, M. D., Texas Medical College, Galveston; Medicine.
DR. ISADORE DYER, Tulane University; Dermatology.
DR. R. H. L. BIBB, Saltillo, Mexico; Foreign Correspondent.
DR. ROBT. MORRIS, Charity Hospital, N. O.; Clinical Reports

The JOURNAL is the official organ of the Austin District Medical Society, the West Texas District Medical Society and the Galveston County Medical Society.

### WHAT A FISH A FROG IS.

Dr. John B. Roberts, of Philadelphia, knows which side his bread is buttered,—and having been born and bred in the city of Brotherly Love, he doubtless thinks it meet that brotherly love (for the homeopaths) should be the means of bread to him at least. He sees very little difference between homeopaths and "Us," as he calls the regular medical profession, and even that little, in the fullness of his heart, he would shut eyes to, and take the whole tribe of Hahnemanniacs to loving embrace; admit them to full fellowship in the Philadelphia-County-and-all-other medical societies. He read an elaborate paper—his Presidential address—before the Philadelphia Medical Society recently, entitled, "Points of Similarity Between Us and Homœopaths," in which he demonstrated, to his own satisfaction, that a frog is a fish of the first water,—lacking only the scales, fins, gills, tail, vertebra, and other minor appendages and appurtenances.

Now, Roberts belongs to that set to whom the Code is a *bete noir*,—a thorn in the flesh,—a real bug-a-boo—and who would revise, or do away with it altogether, so that consultations with homeopaths might be made tributary to the specialty mill. It was a plausible paper—demonstrating a wonderful elasticity of imagination on the writer's part. He could see striking resemblances between "Us" and the Homeopaths in the most dissimilar features,—and there were some present to echo, as did Petruchio's toadies—"Aye, sir, very like a whale."

But Solomon—the synonym for wisdom,—Solomon Solis-Cohen, and others, saw the legendary bug, under the traditional chip, suspected it was only an entering wedge,—a prelude to tinkering with the code, and Solomon was loaded for him, so to speak; Solomon, the "son of his father," according to the irreverent Love (of St. Louis) who is not embarrassed with brotherly love for all kinds of "Us-es," however regular,—demolished poor Roberts with a counter-blast, entitled "Some Points of Dissimilary between Homeopaths and Us." Like his ancient prototype, Sampson, who slew the Philistines with the jaw bone of an ass, he slaughtered this lone Philadelphistine with an apt anecdote of one. Cruel Solomon! Here it is. In opening his paper,—a very excellent one,—in which he used up poor Roberts completely—leaving not "the hair, nor the hide of him," visible, Solomon, after pointing out that all homeopaths, however "liberal," are graduates of *Hahnemann* medical colleges, said:

"I have read somewhere an apologue which, slightly modified to suit the present occasion, runs thus:

"In the country of the Houhnhyms, concerning which land, its inhabitants and their customs, an interesting and reliable account has been left by the late learned and ingenious Gulliver, there was at one time an agitation in favor of what was termed 'liberalization of the Constitution.' In furtherance of this movement there was presented to one of the scientific societies, by an eminent and thoroughbred stallion, a very able paper entitled, 'Points of Resemblance Between Us and Jackmules.'

"With the great generosity and candor, which were its author's characteristics, this paper, while admitting that all mules were not alike, and that the earlier breeds had been quite obnoxious, yet claimed that in recent times a great change had taken place; that, indeed, mules were becoming every day less mulish; and it counted in favor 'of the great body of mules at the present time,' even down to the least fraction of an inch, by which their ears had been shortened, and the last hair in the little bunch at the end of their tails, every point wherein they had never so slightly departed from the anatomy of the more lowly line of their parentage, and approached to the form and features of the noble line that they shared with the speaker. Many of the younger Houhnhyms were moved by his eloquence to respond to the generous impulses of the advocate of fraternization; and it appeared as if his views might prevail. At this juncture a veteran 'stud' arose, and with great gravity and deliberation spoke about as follows:

"'My noble friend has shown most clearly and convincingly that many Jackmules have done their best to imitate Us. Let me even admit, for argument's sake, that he has succeeded in establishing many points of similarity. But he must grant me permission to call attention to one point of difference. There never was a mule yet but he was sired by a jackass.' "

